# Witzenberg Women’s experience of health care after a miscarriage: A descriptive qualitative study

**DOI:** 10.4102/phcfm.v16i1.4581

**Published:** 2024-08-30

**Authors:** Marisa Crous, Ts’epo Motsohi, Adeloye A. Adeniji

**Affiliations:** 1 Department of Family and Emergency Medicine, Faculty of Medicine and Health Sciences, Stellenbosch University, Stellenbosch, South Africa; 2 Education and Training Department, Australian College of Rural and Remote Medicine (ACRRM), Northern Territory, Australia

**Keywords:** miscarriage, early pregnancy loss, emergency centre, rural, qualitative, perception

## Abstract

**Background:**

Although some evidence is available from low- and middle-income countries, no South African data are available on how women experience healthcare during treatment for an incomplete miscarriage.

**Aim:**

This study sets out to explore and describe the experiences of healthcare among women who suffered an incomplete spontaneous miscarriage in the Witzenberg subdistrict, a rural area in the Western Cape province of South Africa.

**Setting:**

Witzenberg subdistrict, Western Cape province, South Africa.

**Methods:**

This study used a descriptive exploratory qualitative study design. In-person interviews were held with women who experienced a miscarriage. Interviews followed a semi-structured format by a single interviewer to explore the various aspects involving experiences of healthcare.

**Results:**

Eight interviews were conducted and analysed. The five themes that arose from transcribed data were: (1) a need for safety, (2) pain management, (3) moderating behaviours and attitudes, (4) disorienting healthcare systems and (5) abandonment. Several factors contributed to the loss of physical and emotional safety in the emergency centre environment. Timeous emotional and pharmacological pain management were found to be a gap while patients awaited care. Clear communication and staff attitude were found to be integral to the patient’s experience and could avoid the perception of abandonment.

**Conclusion:**

There is a universal need for basic respectful, supportive and safe care in patients who attend an emergency centre for early pregnancy complications in rural South African. Specific focus should be given to clear communication and appropriate emotional support during and after the miscarriage.

**Contribution:**

This study can be used as a guide to improve services by ensuring respectful, transparent, informed, and appropriate continuity of care.

## Introduction

Women in sub-Saharan Africa have a miscarriage rate of 0.96% and 1.6%, while the rate in South Africa specifically is 0.96%.^1,2^ In high-income countries like Finland, lower rates of 0.5% are seen.^3^ Miscarriages are defined as a loss of a foetus before 24 weeks of gestation or under 500 g if the gestational age is uncertain.^4,5^ In rural areas of South Africa, women who suffer a miscarriage are often seen and assisted in the Emergency Centre of a district hospital where 24-h medical and ultrasound services are available.^6^ In Ceres Hospital, patients attending for emergency care are all seen in the emergency centre. Only patients with a gestational age above 20 weeks who attend hospital for emergency care are managed in the maternal obstetric unit.

Patients presenting to the emergency centre are prioritised according to the South African Triage Scale, which assesses their acuity or level of urgency.^7^ According to this scale, patients with pregnancy and vaginal bleeding are classified as ‘urgent’, whereas pregnancy and abdominal pain are ‘very urgent’.^7^ Management of these patients is guided by the National Standard Treatment Guidelines and Essential Medicines List for South Africa.^8^

High-income countries have studied the psychological effects of miscarriages on women and their partners. Units dedicated to early pregnancy care in England have been found to contribute to adverse outcomes, where the attitudes of staff were experienced as blunted and routine.^9^ In Canada, where women were treated in busy casualty units, patients were dissatisfied not only with the amount of information given but also with how the bad news was broken.^10^ Even the coordination of care during their miscarriage and discharge was found to be unsatisfactory.^10^ In the United States, women were dissatisfied with the chaos in the emergency room and the lack of privacy.^11^

Although some evidence is available on the distressing and painful experiences of women in low- and middle-income countries (LMICs) who suffered miscarriages, no South African data are available.^12^ Studies conducted in Pakistan and Cameroon have explored the psychosocial experiences of women who suffered a miscarriage, but their views on the medical and surgical care have not been studied.^12,13,14^ In Kenya, health-seeking behaviours of women who had suffered a miscarriage and/or bore a child with a congenital abnormality were explored using focus groups.^15^ However, no data are available on how women perceived healthcare services in LMICs after a miscarriage.

The World Health Organization (WHO) defines good quality healthcare as person centred, equitable, safe and acceptable.^16^ In South Africa, safe and high-quality maternal care is described in the national guideline on maternal care.^6^ Understanding patients’ perceptions of the various aspects of service delivery can guide health services on how to achieve quality care.^17^ Therefore, understanding the experiences from a South African rural perspective is essential to ensuring that district hospital services in these areas meet the specific needs of these patients and are structured to provide a humane and minimally traumatic experience.

The aim of this study was to explore the lived experiences of healthcare among women who suffered a spontaneous incomplete miscarriage in the Witzenberg subdistrict, a rural area in the Western Cape province of South Africa. The objectives of the study were:

- to explore their experience of different health professionals and workers who contributed to their care in the healthcare team, e.g. medical officers, nurses, receptionists, porters and security;
- to explore their experience of receiving bad news from their health professionals;
- to explore the information received, informed consent for and understanding of medical or surgical treatment;
- to explore their experience of the hospital infrastructure and environment, e.g. cleanliness, noise, privacy; and
- to explore the information given and understanding of discharge planning, safety netting and follow-up care.

## Research methods and design

### Study design

This was a descriptive exploratory qualitative study design.

### Study setting

The Witzenberg subdistrict in the Western Cape, South Africa, consists of a diverse population of more than 130 000, according to the 2016 community survey.^18^ Only a quarter of the population completed high school.^18^ Despite the level of schooling and estimated literacy rate of below 60%, the subdistrict has a low level of unemployment of only 6.9% in 2020, which was the lowest in the district.^19^ Ceres District Hospital (CDH) is a government hospital and the referral centre for eight primary healthcare centres (clinics) within the different areas of the subdistrict. The hospital serves mainly patients who do not have medical insurance.^20^ Only 23.7% of people in the Western Cape province are members of medical aid schemes.^20^ The hospital employs two family physicians, and an obstetrician-gynaecologist visits the facility monthly. Clinicians in the emergency unit range from community service medical officers to family medicine registrars. The surrounding small hamlets are situated in the vast 10 753 km^2^ subdistrict, which equates to the lowest population density in the district of 14 people/km^2^.^21^ Patients travel from remote distances to seek help at CDH. The hospital is a 100-bed hospital with an average bed occupancy rate of 91.6%.^22^

### Population and sample

**FIGURE 1:**
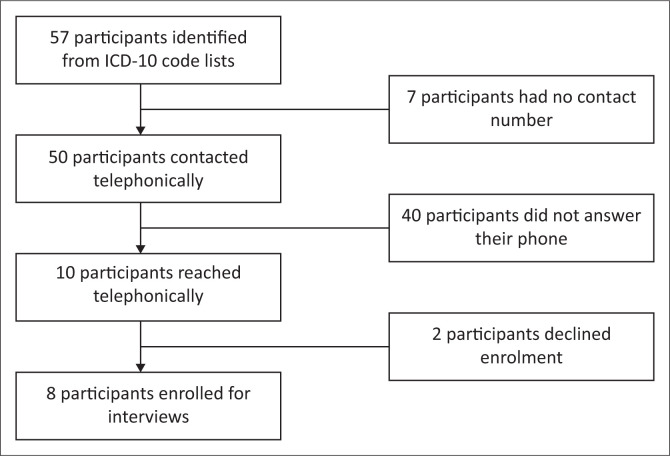
Sampling of participants.

The study population consisted of women over the age of 18 years who suffered an incomplete miscarriage and received treatment at CDH between August 2021 and May 2022. The treatment options were either surgical evacuation in operating theatre under sedation or medical treatment using only misoprostol. Patients with an ectopic pregnancy and gestational trophoblastic disease, who were treated by the researcher or who completed treatment at another facility, were excluded. Participants were purposefully selected from the tenth revision of the International Classification of Diseases (ICD-10) code lists to achieve a diverse group in age, language, parity, treatment and geographical area. The theatre register was used to ensure that all patients treated surgically were present on the ICD-10 code list. Sampling can be seen in Figure 1.

Participants were telephonically contacted and recruited from these lists over a period of 6 months. Participants who did not initially answer their phones were contacted on different days at different times of day. As participants were recruited over time, the selection of future participants from registers became purposeful to ensure an equal distribution of treatment modality, home language and area of residence.

A total of eight participants were interviewed. All participants completed a mental health screening questionnaire prior to the interview. The principal investigator discussed the results of the mental health questionnaire with the participants to ensure that they were aware of possible emotional distress during the interview. Participants were urged to terminate the interview if emotionally distressed.

### Researcher characteristics and reflexivity

The principal investigator was a third-year family medicine registrar at the time of the interviews. She is female, Caucasian and from a different socioeconomic background. She has never utilised medical care for this condition nor has been a patient in this setting. During her previous encounters at primary care facilities, she identified that the care of women experiencing miscarriages was lacking. Family medicine registrars at rural district hospitals participate in governance activities and work in both hospital and primary healthcare centres. The principal investigator has an interest in the experiences of women at Ceres Hospital after suffering a miscarriage but was not the treating clinician for any of the participants. Training in interviewing is included in the postgraduate family medicine programme at Stellenbosch University and prepared the principal investigator for the interviews.^23^ Additionally, guides were used to prepare for interviews.^24^

### Data collection and processing

Semi-structured interviews were conducted one-on-one based on a pre-set interview topic guide. The following questions were used to guide the interviewer when conducting the interview:

- What was your impression of the different members of the healthcare team at Ceres Hospital that you encountered on your journey throughout Ceres Hospital?
- What did you think of and felt about the way that you were told that you had a miscarriage? Is there anything that you think could have been done differently?
- What do you think about the treatment that you received?
- What is your opinion on the hospital’s facilities where you were treated?
- Did the healthcare team make any provisions or follow-up plans on your final appointment at the hospital?
- Is there anything you think could have been done differently throughout your care?

The interviews were conducted 6–9 months after the participant experienced a miscarriage. All participants gave written informed consent in the language of their preference.

Each interview lasted 45 min – 60 min and was conducted by the researcher in either English or Afrikaans. A translator was involved only for isiXhosa- and Sesotho-speaking participants. Interviews were recorded with two separate recording devices and saved on both an electronic cloud and a hard drive that was stored in a passcode-protected safe. All informed consent documents and field notes were electronically saved and stored in the same manner as the recordings. Electronic copies, recordings and transcripts were stored on a secure password-protected, cloud-based platform. Participant anonymity was maintained throughout the process by removing identifiers proceeding with only an assigned number.

Interviews were conducted by the researcher at the local clinic and not at the hospital to ensure that the environment is as neutral and convenient as possible for the participants. Each participant was provided with a shopping voucher with the value of (ZAR) 180 for their time, inconvenience and expenses. No participants were harmed or experienced emotional distress during this study. Interviews were conducted until no new information was obtained (operational saturation). This occurred after six interviews, and the last two interviews were conducted to confirm this saturation.

After the transcripts were generated, the participants were given the opportunity to read their transcripts. Five participants collected their transcripts, and all five were satisfied that the transcripts were true reflections of the interviews. One participant declined to read the transcript and the remaining two were not contactable. Of the participants who read their transcripts, none had any additional comments to add.

### Data analysis

The framework approach was used to analyse the data from the interviews.^25^ The recordings were translated and transcribed by professional services, and the following steps were then followed by member checking:

- **Familiarisation:** The principal investigator read each transcript and checked it against the original recording. Key issues requiring coding were noted.
- **Coding:** Based on the different study objectives and questions described earlier, data sections were coded during immersion in the data through repeated reading and review of the transcripts.
- **Indexing:** A coding index was developed from the codes applied to the data. ATLAS.ti was used to handle the indexing of the transcripts (version 23.0.8.0) (http://www.atlasti.com/).
- **Charting:** Data from the same coding index were aggregated for interpretation. These were refined through discussion with the second researcher (TM).
- **Interpretation:** Each chart was reviewed by the researcher to identify themes and subthemes. These were discussed and debated with the second researcher (TM) until consensus was achieved.

### Ethical considerations

This study was approved by the Health Research Ethics Committee (Medical) at Stellenbosch University (Reference: S21/07/145) as well as the Western Cape Department of Health (Reference: WC_202201_023).

### Trustworthiness

All participants had the personal experience of receiving treatment for an incomplete miscarriage at Ceres Hospital. The principal investigator personally checked the translated transcripts against the original recording. Member checking was conducted. A description of the demographics of the study population is given in Table 1 to aid with transferability. The second researcher (TM) read through all the interviews and noted key issues. These key issues were checked against the coding of the principal investigator to ensure dependability. In addition, extensive notes and annotations of the transcripts were kept and stored to assist with an audit trail. To assist with credibility and bracketing, the second researcher (TM) kept the principal researcher aware of a priori assumptions and beliefs while interpreting the data, and these were discussed reflectively.

**TABLE 1:** Participant sociodemographics (*N* = 8).

Participant characteristic	Number of participants
**Treatment type**	
Surgical	4
Medical	4
**Home language**	
Afrikaans	5
Sesotho	1
isiXhosa	2
**Relationship status**	
Single	2
Married	3
Divorced	1
Committed relationship	2
**Employment status**	
Employed	3
Unemployed	5

Lastly, the researcher reflected on each interview following the interview and made notes to adjust the interviewing style.

## Results

Eight women participated in this study and were from different areas within the subdistrict. Their ages ranged from 19 to 42 years and the group had an equal division of surgically and medically treated incomplete miscarriages. The median age was 36 years, and the mean age was 33 years. More characteristics of the group can be found in Table 1. Five themes emerged from the semi-structured interviews.

### A need for safety

The need for safety emerged in different forms throughout the interviews. Participants expressed fear about their safety in the emergency centre environment where undifferentiated patients are admitted to a general area. They expressed reluctance even to sleep because of fear of other patients:

‘I was scared because this woman even looked wild, had wild behaviour, walking up and down. She also grabbed an old man by his chest … and a man came in, a young guy that was assaulted; he was acting out on the floor.’ (Participant 2, G6P2M4, Married)

‘There was somebody that was mentally disturbed. He kept undressing the bottom part of his body and got into bed with a woman in the women’s ward, got in behind her back.’ (Participant 4, G3P2M1, Married)

Participants also yearned for the safety through respectful care and protection of confidentiality. Nursing staff would announce the diagnosis and treatment plan for each patient in an open area on handover rounds:

‘Every Sister [*professional nurse*] that came on shift [*said*]: This one had a miscarriage; their medication is this and this. This one must sit. The Sister saw to us in that way.’ (Participant 3, G3P1M1, Committed relationship)

‘I felt bad then [*when diagnosis announced in front of everyone*]. Then I said, “I am not worried. I just want to leave here. If I can just leave this place, then I will be satisfied.”’ (Participant 3, G3P1M1, Committed relationship)

Participants voiced that they were examined in areas where the barriers did not allow for the protection of their privacy. They expressed the need to be admitted to an area separately from other patients to save themselves from embarrassment:

‘Now, it is those curtains that cannot even close properly. And I thought to myself, no, doctor, can you not take me into the other room, like the one the doctor took me to on Thursday, and closed it? I told her to ensure that it was properly closed. And while she was examining me a nurse peeked in. There I am lying totally uncovered, and the nurse peeked in.’ (Participant 6, G2P1M1, Married)

‘I was bleeding through, I was thinking and aware and just must sit there, the sanitary pad isn’t going to work. Then I asked a cleaner that I’m bleeding badly, she’ll have to come clean up and she did and brought me other clothes and cleaned up. But every time you sit, you feel you can’t sit amongst those people.’ (Participant 5, G3P2M1, Divorced)

### Pain management

Emotional and physical pain played a significant multidimensional role in the experiences of the participants. Some participants complained that despite informing healthcare staff of their pain and requesting analgesia, it was still refused on the premise that only the doctor could prescribe analgesia. To some participants, this meant that they had to endure physical pain for extended periods:

‘She [*the nurse*] then advised me to wait in the waiting area. And yes, I waited. Later the pain became worse and pieces came down. I then went to her and asked for pain medication, and she told me that I should just wait for doctor, and doctor only came the next morning.’ (Participant 1, G2P1M1, Single)

Other participants felt that they needed to display the severity of their pain to receive help or before they had received any attention to their physical pain:

‘I sat there and this pain started getting worse and worse and worse and worse … I was on the floor, crying and oh, it was too much. It was too much.’ (Participant 8, G2P0M1, Committed relationship)

‘A young guy that was assaulted; he as acting out on the floor, he was in pain, they must help him. We still weren’t helped, so they helped him with tablets. So, I said it seems one must cry and perform before you can get helped.’ (Participant 2, G6P2M4, Married)

The management of emotional pain proved as important as alleviating physical pain. For participants who had experienced emotional pain because of this uncertainty about their health situation, finally being informed of the outcome provided relief. Participants expressed a need to engage in conversations about fertility, their related health concerns or options for further psychological support – stating that it could have lessened emotional pain. Some participants noted that the discharge plan healthcare workers had verbally mentioned to them did not reflect upon their actual discharge:

‘I thought that perhaps they would send me to the clinic afterwards, perhaps tell me … I did not think that everything would stop right there at the hospital.’ (Participant 4, G3P2M1, Married)

‘Because I had many questions, do you understand doctor? I had to go home with all the questions, and I had to be strong. I had to, not “calm myself,” but I didn’t feel sorry for myself, I expressed my feelings at home, and I had to make myself feel better with the words.’ (Participant 1, G2P1M1, Single)

### Moderating behaviours and attitudes

Negative experiences outweighed positive experiences when participants discussed their interactions with various healthcare workers during their hospital stay. Participants expressed a need for being cared for and attended to with respect and kindness:

‘I see the doctor as someone who does not respect their work. Because if she or he respected his work maybe they will treat [*me*] in a better way.’ (Participant 6, G2P1M1, Married)

However, accounts of unprofessional behaviour were heard from some of the participants. They reported being scolded and belittled when they did not adhere to instructions given by the healthcare workers. Participants also admitted to personal judgement regarding their age, financial capacity, race and choice to conceive:

‘And he told me that don’t I know that I don’t have to have sex when I’m pregnant, and I told him that I didn’t know I was pregnant. But he just gave me the attitude […] It felt to me like they were judging me because I’m pregnant and I had a miscarriage.’ (Participant 8, G2P0M1, Committed relationship)

‘The one sister who did the observation, she wanted to get nasty, then she said, in a very nasty way, “in the month of August, you had a miscarriage and went and made funny business and now you’re here again with a miscarriage.” Because I did tell her, it’s not as if she’s working for my children. As if I’m going to ask her something if I’m in need, I work for my own children.’ (Participant 2, G6P2M4, Married)

‘That’s why I say, people from here, from Western Cape, they choose the face. If it were somebody else, they would have made the effort to make an appointment. Pap smear would be taken. All those things. If you are well-known, then they would have done those things. Now, I am not well-known. I am just black person.’ (Participant 3, G3P1M1, Committed relationship)

A break in trust developed between the hospital and participants in cases where they experienced negative behaviour from staff. Some participants were even reluctant to return to the hospital for future health concerns and were still experiencing a trauma response when in contact with the hospital:

‘Still now I get a fright when the hospital phones me.’ (Participant 3, G3P1M1, Committed relationship)

‘What makes me angry and hurts me, is just that piece; you sit on that chair. They come to you whenever they feel like for you to come to see you […] Even now, I wish if I could go and give birth I just go home. I do not feel like that hospital. Or I should go just for my file; they should transfer me to, for example, Worcester, or another hospital, but not this hospital.’ (Participant 3, G3P1M1, Committed relationship)

‘I said, “I pray every day: I don’t want to sit at that hospital again because you don’t get the treatment that you should get there.”’ (Participant 2, G6P2M4, Married)

In contrast to the negative experiences, other participants felt free to return to both the hospital and related clinics when they experienced a caring practitioner. Some participants gave accounts of healthcare staff who went beyond their duties to ensure that the patients were emotionally and physically cared for. This was done through the regular review of a participant’s level of pain and comfort:

‘He [*the doctor*] helped me very nicely. And he told me that I must not feel bad that this happened. It does not happen with everybody, but it can happen to anybody. So, he said it is not the first time that he has encountered a case like this. For him it is something that is painful, but he said that he would not understand how the next person would feel who was going through this.’ (Participant 7, G2P0M2, Single)

‘What did the doctor say now? But the doctor did tell me that within a certain time that I must come back to the clinic, so that they can check whether everything is normal inside me. That everything has moved back into place. He still told me how long it takes when everything has moved back into place again. Yes, the doctor did say, but I have never … I just made the appointment for me with Sister. And I just never came back so that they can check.’ (Participant 7, G2P0M2, Single)

### Disorienting healthcare systems

‘I was not prepared, I did not know what to expect, this was my first time.’ (Participant 1, G2P1M1, Single)

More than one of the participants mentioned that because it had been their first time experiencing a miscarriage, they did not know what to expect of the hospital environment, staff or of aspects of their management plan. Even an investigation like ultrasound was startling to a patient who had not been prepared for it:

‘Maybe [*they*] could have told me that they were going to do a second sonar. That they were going to push the sonar up my buttocks because I did not know. Because I thought that they would do the same kind of sonar as before, because I was lying down. Then I suddenly felt that something was being pushed in by my buttocks, and it was very uncomfortable and sore.’ (Participant 7, G2P0M2, Single)

Participants expressed that they were told to wait in hospital without knowing what they were waiting for or were given treatment with little explanation as to the indication. A participant mentioned the colour system of the South African Triage Score system but still expressed concern over the prioritisation of her complaint:^7^

‘I can’t remember now how it’s written, less dangerous or get more urgent attention or… but it is like that. For me it sounded, my case is not so urgent, and she [*the nurse*] said “Go put my file in the green or orange basket.”’ (Participant 2, G6P2M4, Married)

‘Then I waited again for about two days there. Don’t know why, but I was there.’ (Participant 8, G2P0M1, Committed relationship)

Participants arriving at hospital resorted to asking auxiliary workers like cleaners or security guards for help while waiting on a system that they are unfamiliar with because they were hesitant to approach healthcare workers. They expressed that certain follow-up plans were mentioned by healthcare workers but not followed through upon discharge. Participants mostly complained that the last healthcare worker that they interacted with were nursing staff upon discharge, missing the opportunity to interact with a doctor about any unanswered questions or follow-up plans. Most participants expressed a need for counselling either in hospital or at the clinic with the aim of clarifying certain health-related queries:

‘I thought that […] perhaps they will give me a letter at the clinic, okay, there someone will counsel you, or there someone will take it further with your, and so, but nothing.’ (Participant 4, G3P2M1, Married)

‘I was told to talk to a doctor because they spoke of a tablet they would recommend, to fall pregnant quickly again as I’ve been cleaned. They say you can fall pregnant again quicker once you’ve been cleaned inside. They haven’t said anything further about the tablet I can take or what. Just gave me the pain tablets again and afterwards. I haven’t as yet seen a doctor again to discuss around that. He mentioned the tablets name that I can take for, because I’m already struggling to fall pregnant again, then he told me he can recommend me a tablet and he hasn’t given the tablet yet.’ (Participant 2, G6P2M4, Married)

### Abandonment

The theme of abandonment manifested across the whole journey of care: from arriving at the hospital to exiting the facility either having to return for a follow-up appointment or having to return home with a myriad of uncertainties and concerns:

‘[*I felt*] like no one cares … like no one cares.’ (Participant 8, G2P0M1, Committed relationship)

‘I was waiting for this opportunity: [*to show*] that there’s caring for us women, who have gone through this because […] many women feel deserted and have no one to talk to.’ (Participant 5, G3P2M1, Divorced)

In contrast to the feeling of being emotionally deserted, participants also expressed a feeling of physical abandonment. One of the participants noted that the doctor repeatedly left during the consultation to attend to emergency cases in other units, leaving her to wait long periods without intermediate or definitive care:

‘Then later the afternoon she [*the doctor*] called me in again. She then wanted to examine me. She told me to sit on the bed. And while I was sitting on the bed she was again called into theatre, and she left me there. And then … It was during a weekend. Then somebody came in that was much more ill, an elderly lady, and I had to vacate the bed and go sit on a chair again.’ (Participant 4, G3P2M1, Married)

Some participants were deserted even in the prehospital setting. Despite calling the Emergency Medical Services, they had to endure long waiting times or even travel with public transport as emergency services never arrived:

‘We phoned the ambulance, say about three times, Friday night till twelve o’clock, nine o’clock in the evening. The ambulance did not arrive. That morning I slept. After that blood clot that fell, then I looked, and saw there is no blood. So, I thought it was over, but in the evening when I lay down, I felt that the blood was still coming out, I got up from my bed and looked in the bed whether I wet the bed. So, I stood up. Washed myself. We took a taxi with my boyfriend, to the hospital. A Taxi!’ (Participant 3, G3P1M1, Committed relationship)

## Discussion

Five themes were identified during this study: (1) a need for safety, (2) pain management, (3) moderating behaviours and attitudes, (4) disorienting healthcare systems and (5) abandonment.

Physical and psychological safety within the emergency centre environment in CDH affected how women experienced the care they received during their stay. The contribution of the emergency centre environment to patients’ perception of healthcare is a well-researched topic. However, the relationship between safety and the emergency centre environment is not well described. Punches et al. describe the emergency centre environment as being ‘chaotic’, and Baird et al. describe it as ‘unfriendly’, but neither study discusses the implication on patient’s perception of safety.^11,26^ Due et al. only describe a ‘bustle’ in the emergency centre but relate this only to decreased individualised care with no discussion or analysis of safety.^27^ Studies addressing patient safety only focus on overcrowding and the resulting decreased quality of care and not on psychological and emotional safety.^28,29,30^

Many participants noted that they were admitted to a chair in the unit to wait for a bed. The open space affected the perception of their own physical safety, as behaviourally disturbed patients were admitted to the same area. This was similar to research by Bull et al. who also described participants’ experiencing other patients impeding on their perceived safety within the emergency centre.^31^

These open spaces, bed shortages, inadequate physical barriers between patients and the behaviour of nursing staff and doctors lead to compromised privacy and confidentiality. Studies specifically focussing on nursing handover at the bedside had comparable results because this study population also noted the importance of maintaining confidentiality.^32^ Participants were left feeling unsafe to engage with staff about their health concerns at times.

Delay in pain management is associated with overcrowding, workload, patient placement and throughput.^29,33^ In our study, some nursing staff refused analgesia because a doctor needed to prescribe it, but did not attempt to liaise with the doctor for a prescription in the interim. Instead, they told participants that the doctor was busy. Providing simple analgesia, such as paracetamol, is within the scope of a professional nurse without the instruction of a doctor.^34^ This may be because of numerous factors, including that nursing staff is being overwhelmed by heavy workloads. In fact, Muluadam et al. identified workload, years of experience, lack of pain management protocols and overcrowding as some of the barriers to effective pain control in an Ethiopian emergency centre.^35^ In our study, participants were left feeling as if they needed to display their physical pain to be helped.

Emotional pain caused by the experience of a miscarriage is universally experienced.^36^ Poor psychological support in the emergency centre and upon follow-up was experienced even in higher-income countries.^11,26,27^ In a randomised control trial by Johnson et al., grief interventions immediately after a miscarriage were found to promote women’s ability to overcome the experience.^37^ Participants in a study by Due et al. experienced worsening distress when left with uncertainties about their health after experiencing a miscarriage, much like some of the participants of our study. Evidence shows that this leads to longer recovery from the emotional aspect of a miscarriage.^27^

The accounts of verbal abuse, discrimination, racism and neglect in our study were similar to those from a study by Asefa et al., who found that abusive behaviour experienced by patients admitted for maternal care originated from high workload, poor support from management and the discomfort with the physical work environment.^38^ In contrast to negative experiences, staff who were perceived as caring in similar studies were appreciated by participants.^11,26^ When patients were provided with sufficient information and involved in their own care, participants perceived these staff as caring. These various attitudes and behaviours clearly played an important moderating role.^26^

When participants were asked to describe the different steps that they followed during their stay at Ceres Hospital, there were numerous accounts of moving from one healthcare worker to the next without understanding their distinct roles. Only some participants were aware of the triage system but appeared confused about what level of prioritisation they had been assigned. This theme of a disorienting health system seems mostly rooted in vital communication gaps. In fact, numerous studies have shown that patients show appreciation and experience decreased levels of anxiety when they were informed about waiting times, movement in the emergency centre and upcoming consultations.^31^ This sentiment overlaps with our study population’s wishes for concrete management plans, including reasons for admission, indication for medication and a structured discharge plan with an appointment booked at the local primary healthcare facility.

The values of Western Cape Department of Health include ‘care’ and ‘respect’.^39^ Lockett et al. recently conducted a similar study at a metropolitan district hospital in the Western Cape and found that the participants in the metropole expressed needs for a supportive and private environment, empathy and caring staff.^40^ In this rural setting, participants also felt physically and/or emotionally abandoned when the services were not experienced as caring and respectful. As previously mentioned, feeling cared for and heard has been shown to assist with the grieving process and ease the experience of a miscarriage in the emergency centre.^27,41^

## Limitations

There are important limitations of this study to note. The research was conducted at only one rural district hospital in South Africa, a country with more than 40 districts.^42^ The researcher is a service provider at CDH and surrounding primary care facilities. Although she was not directly involved in the care of these patients, her positionality in this regard could have affected the engagement with participants. Bracketing could have been improved if the researcher was interviewed herself and was able to reflect on the responses to questions. More reflexive debriefing sessions throughout the data collection would have also improved the confirmability of the findings.

The diagnosis of an incomplete miscarriage was based only on ICD-10 coding. No file reviews were done at CDH to determine how the diagnosis was made.

The researcher interviewed in English or Afrikaans and required a translator for interviews in isiXhosa and Sesotho. This means that a level of thickness may have been lost in translation. Forty participants could not be contacted using the telephone number in their medical record. Possible reasons for this include poor cellular phone reception in rural areas, incorrect contact numbers, changed contact details or participants not answering a call from an unknown telephone number. Not all areas within Witzenberg were included because participants were not contactable or declined participation. Participants utilised only public health services for the treatment of their miscarriage, and findings cannot be generalised to the experience of private medical care in Witzenberg.

Despite these limitations, the findings are mostly consistent with other studies investigating this topic, as themes about respectful and confidential care are universal. The nature of qualitative studies such as this is that most findings cannot be generalised. However, many of the findings could be used to inform and focus on a broader quantitative study that covers more facilities.

## Conclusion

This study provided a unique and unprecedented perspective on the strengths and weaknesses of care provided to women who have received public healthcare for miscarriages in rural South Africa. It highlighted that there is a universal need for basic respectful, supportive and safe care in patients who attend an emergency centre for early pregnancy complications in a rural South African setting. The authors recommend that this research be used to guide policymakers to improve services by ensuring respectful, transparent and informed care, pain management and appropriate continuity of care throughout the system’s pathways. Specific focus should be given to appropriate emotional support during and after the miscarriage.
